# Na_2_SeO_3_ Exposure Inhibits Locomotion and Reproduction via Oxidative Stress Mechanism in Bioindicators *C. elegans* and *Acrobeloides* sp.

**DOI:** 10.3390/toxics13100870

**Published:** 2025-10-13

**Authors:** Fan Jin, Jiaping Song, Shanshan Niu, Xuebin Yin

**Affiliations:** 1 College of Resource and Environment, Anhui Science and Technology University, Fengyang 233100, China; 15755009328@163.com; 2 Institute of Functional Agriculture (Food) Science and Technology at Yangtze River Delta, Anhui Science and Technology University, Chuzhou 239000, China; 3 Anhui Province Key Laboratory of Functional Agriculture and Functional Food, Anhui Science and Technology University, Chuzhou 239000, China; 4 School of Earth and Space Sciences, University of Science and Technology of China, Hefei 230026, China; nss@mail.ustc.edu.cn

**Keywords:** selenium, nematode, bioindicator, *Caenorhabditis elegans*, oxidative stress

## Abstract

Selenium (Se) fertilizers are widely used in Se biofortification. However, excessive application of Se fertilizers can easily cause soil Se accumulation and threaten soil organisms, so it is necessary to carry out research on the effects of Se on single species, especially on indigenous organisms in soil. In this study, as significant bioindicators, *Caenorhabditis elegans* and *Acrobeloides* sp. were used as research subjects to explore the effects of short-term Se exposure on the total Se content, Se speciation, and physiological and biochemical functions in nematodes. The results showed that total Se content in both nematodes increased significantly with higher Se exposure concentrations, and Se mainly exists in the speciation of selenomethionine (SeMet) and selenocysteine (SeCys_2_). Exposed to high concentrations of Na_2_SeO_3_ (≥3 mg/kg), *Caenorhabditis elegans* and *Acrobeloides* sp. were strikingly inhibited in their locomotion and reproduction, and oxidative stress response was induced, which showed a significant increase in ROS, MDA, and SOD. Pearson correlations suggested a depressant effect on nematodes by oxidative stress mechanism. This study provides an important basis for the soil ecotoxicity assessment of Se and highlights the unique value of *Acrobeloides* sp. in ecotoxicological studies.

## 1. Introduction

Selenium (Se) is an essential trace element for humans and animals, and plays an important role in antioxidant defense, immune regulation, and metabolic processes [1,2]. Therefore, Se-enriched fertilizers have been widely used in the production of Se-enriched agricultural products. However, the beneficial effects of Se are very narrow [3], and moderate amounts of Se are good for organisms, while excessive contents of Se may trigger toxic effects that can lead to oxidative stress, cell damage, and even death. With the popularization of Se biofortification technology in agriculture, the application of Se fertilizers may lead to excessive accumulation of Se in the soil, which can endanger soil organisms. The high concentration of Se accumulated in animals’ bodies will compete with the sulfur element and replace it in the amino acids, disrupting the expression of protein in the body, and causing damage to tissues and organs [4]. Furthermore, selenite and diselenides can react with oxygen in the presence of thiols to produce superoxide cycles, cell cycle arrest, and endoplasmic reticulum stress reactions in animals [5]. Evaluating the ecotoxicological effects of Se on soil organisms can provide a scientific basis for the development and utilization of Se-enriched soils and the management of Se application in agriculture, thereby protecting the health of the soil ecosystem.

As the most abundant metazoan in the soil, nematode plays a crucial role in matter circulation and energy flow [6], which is an ideal model organism for evaluating soil ecotoxicology [7]. Se can directly change the number and community structure of soil nematodes [8], while the direct effect of Se on nematodes and its mechanism are still unclear. Standardized toxicity testing is a suitable tool for assessing the bioavailability and environmental effects of trace elements. The sensitivity of cultivated nematodes makes them effective for evaluating the impact of chemicals on soil ecosystem health in exposure experiments.

The model animal of nematode *Caenorhabditis elegans* (*C. elegans*), a free-living nematode with the abundance in composts and decaying plant material, has the advantage of short life cycle, easy culture, clear developmental and genetic background [9,10], and has been widely used in ecotoxicology study [11,12]. Studies using *C. elegans* as a model have shown that low Se concentration has protective effects on the nematodes, and can promote the reproduction and development of nematodes, while high Se concentration has negative effects that can lead to reproduction and motor damage [13,14]. The ecotoxic effect of Se on *C. elegans* may be caused by oxidative stress mechanism. Among tested nematodes, *C. elegans* has been the focus of extensive research, with multiple endpoints compared [15,16,17]. However, various nematode species may have different sensitivity to toxicants due to differences in behavioral, morphological, and life history characteristics. Furthermore, *C. elegans* is usually cultured artificially and is relatively uncommon in soil [18], so studies using it as the experimental subject are not enough to evaluate the effects of Se on soil nematodes. *Acrobeloides* sp. was widely used in the exposure studies of heavy metals and organic matters because of its indicative nature [19,20,21,22]. The study showed that when compared with the low and medium Se treatments, the relative abundance of *Acrobeloides* sp. in the soil of the high Se treatment decreased, and there was a significant negative correlation with the total Se contents in soil, indicating that *Acrobeloides* sp. may be highly responsive to Se [23].

Currently, most of the studies on the toxic effects of Se on nematodes focus on the total Se content, survival rate, and lifespan, but the distribution of Se species in nematodes and the mechanism of its influence on physiological functions are still not clear. In addition, the toxic effect of Se on nematodes through oxidative stress mechanism still need to be further verified. Therefore, we conducted a study in which sodium selenite was used as the Se source, and *C. elegans* and *Acrobeloides* sp. were exposed to different concentrations of Se in nematode growth medium (NGM). At the same time, the accumulation of Se in two nematodes was analyzed, and the toxic effects and mechanism of Se on nematodes were revealed from the aspects of nematode reproduction, locomotion, and antioxidant indexes, which provided a scientific basis for evaluating the soil ecological risk of Se.

## 2. Materials and Methods

### 2.1. Reagents

Sodium selenite [Na_2_SeO_3_, Se(IV)] and chemicals were purchased from Shanghai Test-Sinopharm Chemical Reagent Co., Ltd. (Shanghai, China) and Sigma-Aldrich (St. Louis, MO, USA), unless stated otherwise. Kits used to determine antioxidant indicators were purchased from Abcam (Cambridge, UK).

### 2.2. Test Nematodes

In this study, the model organism *C. elegans* and indigenous nematode *Acrobeloides* sp. were selected as subjects, and both nematodes take OP50 as food. *C. elegans* is a common model organism with a 3.5 day reproductive cycle and a 30 day lifespan. *Acrobeloides* sp., a soil free-living nematode was isolated from pristine soil and preserved after purification and sterilization. It has a reproductive cycle of 10 days and a lifespan of 80 days [20]. These two purified nematodes and *Escherichia coli* (*E. coli*) OP50 were provided by the research group of Professor Li Huixin from Nanjing Agricultural University.

### 2.3. Nematodes Maintenance and Treatments

Before the exposure experiment, the nematodes were grown on NGM in a biochemical incubator at 20 °C [24], and gravid adult nematodes were subsequently synchronized by lysis method [21,25]. After that, the synchronized L4 stage larvae could be obtained and be used for the following experiments. The prepared aqueous solutions of selenite was added to sterilized NGM cooled to approximately 60 °C, achieving the Se concentration of NGM to 0, 0.4, 3, 5, 10 mg/kg (with reference to standard values for Se content in naturally Se-enriched and Se-excessive soils), respectively, and three parallel samples were set up for each treatment group. The mixture was poured into Petri plates and allowed to solidify, which were then seeded with *E. coli* OP50 for the subsequent nematode exposure experiments.

### 2.4. Analysis of Se and Oxidative Stress in Nematodes

A large number of synchronized L4 nematodes were seeded onto centrifuge tubes with 0.5 mL of M9 buffer. The supernatant was removed by centrifugation, transferred to Petri plates containing *E. coli* OP50, and placed in a biochemical incubator for growth at 20 °C. *C. elegans* and *Acrobeloides* sp. were cultured for 5 days, washed 3 times with M9 buffer, resuspended in 2 mL of M9 buffer and homogenized on ice. The homogenate was centrifuged at 2500 r/min for 10 min, the supernatant was taken, and the protein content was determined with the BCA (Bicinchoninic Acid Assay) kit.

#### 2.4.1. Total Se Quantification in Nematodes

The total Se content of nematode homogenate was detected by AFS (Atomic Fluorescence Spectrophotometer), and the Se content of nematodes was calculated by combining with the protein content of the supernatant of the homogenate. The calculation formula is as follows:

Total Se content in nematodes: C_0_ (μg/mg protein) = C_1_/C_2_ (1)

where C_1_ is the total Se content of the homogenate and C_2_ is the protein content of homogenate supernatant.

#### 2.4.2. Se Speciation in Nematodes

HPLC-ICP-MS (High Performance Liquid Chromatography-Inductively Coupled Plasma Mass Spectrometry) was used for the analysis of the standard solution and the aqueous extraction [26]. Selenocysteine (SeCys_2_), Selenomethionine (SeMet), Methylselenocysteine (SeMeCys), selenite (Se^4+^) and selenate (Se^6+^) were quantitatively analyzed by area normalization method, and the contents of Se speciation in nematodes were calculated by combining with the protein content of the supernatant of the homogenate. The calculation formula is as follows:

Contents of Se speciation in nematodes: S_i_ = S_1_/C_2_ (2)

where S_i_ is the contents of i-th Se speciation, S_1_ is the content of Se speciation in the homogenate, and C_2_ is the protein content of homogenate supernatant.

#### 2.4.3. Determination of Oxidative Stress in Nematodes

The obtained nematode homogenate with known protein content was determined by using ELISA kits for superoxide dismutase (SOD) activity (cat. no. ab65354; Abcam, Cambridge, UK), glutathione peroxidase (GSH-Px) activity (cat. no. ab102530; Abcam, Cambridge, UK), reactive oxygen species (ROS) level (cat. no. ab139476; Abcam, Cambridge, UK) and malondialdehyde (MDA) content (cat. no. ab118970; Abcam, Cambridge, UK).

### 2.5. Analysis of Locomotion and Reproduction

#### 2.5.1. Body Activity Frequency

Fifty synchronized L4 nematodes were seeded onto NGM containing *E. coli* OP50, and placed in a biochemical incubator for growth at 20 °C. On the first and the seventh day of the exposure experiment, three nematodes were randomly selected on each NGM to record the twisting times of the nematodes’ body within 1 min, that is, the activity frequency. Given that the nematodes were traveling along the *x*-axis, body activity frequency was identified by a change in the direction of the posterior pharyngeal bulb’s movement along the *y*-axis [27].

#### 2.5.2. Reproductive Profile

Five synchronized L4 nematodes were seeded onto NGM containg *E. coli* OP50, and placed in a biochemical incubator for growth at 20 °C. The inoculated nematodes were transferred to another identical Petri plate every day, and the newly hatched nematodes in the original dish were counted under a microscope. After three days, the total number of hatched nematodes in three days was calculated, divided by the number of inoculated nematodes; this was recorded as the number of eggs laid.

### 2.6. Statistical Analysis

All data are expressed as means ± standard deviation (SD) for each experimental group. Statistical analysis was performed using SPSS 25.0 software and R language 4.0.5, and Origin 2021b was used for data visualization. One-way variance and Tukey test were used to analyze the significant differences in various indexes of different Se exposure concentrations. Pearson Correlation Analysis was used to verify the correlation among indicators. Parametric non-linear regression fitting was used to quantitatively analyze the relationship between Se exposure and physiological indexes of nematodes.

### 2.7. Method Validation

The certified reference material SELM-1 (Selenium Yeast) was used as a standard for selenium amino acid speciation. The recovery rate of SeMet fell within the acceptable range of 80% to 120%. The limits of detection (LODs) for the five selenium species were as follows: 0.52 ng/mL for Se^4+^, 1.51 ng/mL for Se^6+^, 1.12 ng/mL for SeCys_2_, 1.81 ng/mL for SeMet, and 1.20 ng/mL for SeMeCys. Calibration curves for each Se species and representative chromatograms are provided in the Supplementary Material.

## 3. Results

### 3.1. Total Se Content and Distribution of Se Speciation in Nematodes

Following a five-day cultivation on NGM with different Se concentrations, the Se contents in *C. elegans* and *Acrobeloides* sp. are shown in Table 1. In the culture medium of the control group, the Se content in *C. elegans* and *Acrobeloides* sp. was 0.06 ± 0.01 μg/mg protein and 0.34 ± 0.15 μg/mg protein, respectively. The Se content in these two nematodes increased gradually with the growth of Se concentration. When the maximum Se concentration was 10 mg/kg, the Se content in *C. elegans* and *Acrobeloides* sp. reached 5.70 ± 2.63 μg/mg protein and 15.26 ± 2.91 μg/mg protein, respectively, which was 95 and 45 times of those nematodes in control group. In addition, it can also be seen from the table that the Se accumulation ability of *Acrobeloides* sp. was stronger than that of *C. elegans*, and the Se content of *C. elegans* in the control and all experimental groups was lower than that in *Acrobeloides* sp.

**Figure 1:**
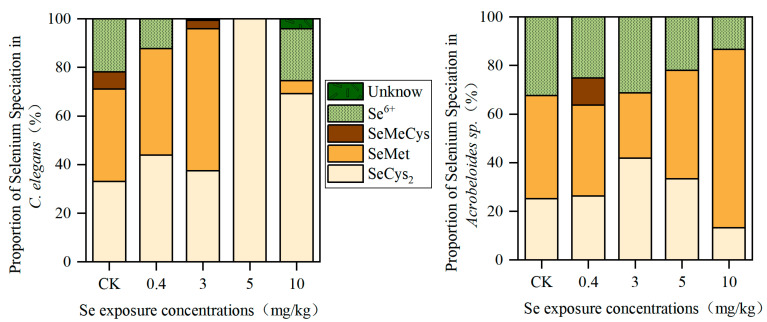
The Se speciation in *C. elegans* and *Acrobeloides* sp.

The distribution of Se species in the two species of nematodes is shown in Figure 1. In the control group, the distribution of Se species was highly similar between *C. elegans* and *Acrobeloides* sp., primarily consisting of SeCys_2_, SeMet, and Se^6+^. When Se concentration was no more than 3 mg/kg, SeMet was the main species of Se in *C. elegans*, accounting for 38.13%~58.55%, followed by SeCys_2_, accounting for 33.04%~43.86%, while SeMet and SeCys_2_ were the main Se species in *Acrobeloides* sp., accounting for 26.83%~42.42% and 25.18%~41.90%. When the Se concentrations went up to 5 mg/kg and 10 mg/kg, SeCys_2_ was the main Se species in *C. elegans*, accounting for 100% and 69.28%, respectively, whereas SeMet was the main Se species in *Acrobeloides* sp., accounting for 44.6% and 73.41%.

### 3.2. Effects of Se on Physiological Indexes of Nematodes

**Figure 2:**
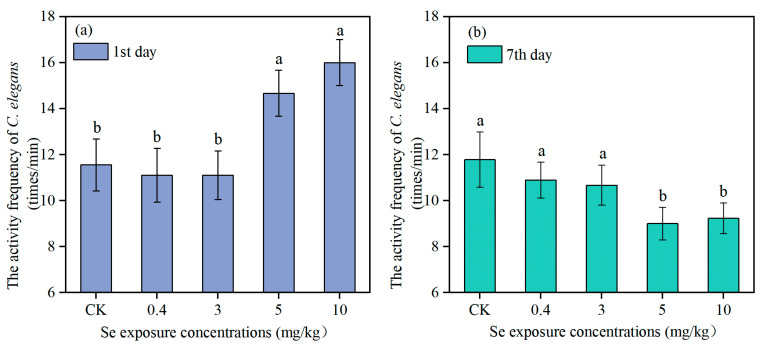
The activity frequency of *C. elegans* under Se exposure (*n* = 3). The times were first day (**a**) and seventh day (**b**), respectively, and different lowercase letters (a, b) indicate significant differences between groups (*p* < 0.05).

**Figure 3:**
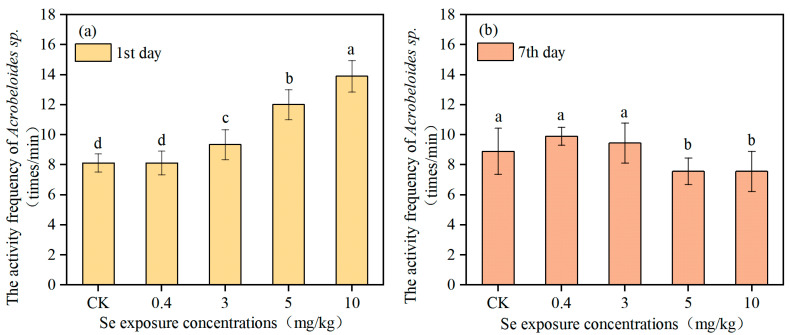
The activity frequency of *Acrobeloides* sp. under Se exposure (*n* = 3). The times were first day (**a**) and seventh day (**b**), respectively, and different lowercase letters (a, b, c, d) indicate significant differences between groups (*p* < 0.05).

Figure 2 and Figure 3 show the activity frequency of *C. elegans* and *Acrobeloides* sp. on the first and the seventh day of Se exposure, respectively. The activity frequency of *C. elegans* on the first day in the control group was 11.56 ± 1.13 times per minute. The lower levels of Se (0.4 mg/kg and 3 mg/kg) had no significant effect on nematode activity frequency, while the activity frequency of *C. elegans* was significantly increased to 14.67 ± 1.00 and 16.00 ± 1.00 times per minute when the Se concentration reached 5 mg/kg and 10 mg/kg. On the seventh day, there was no significant change in activity frequency of *C. elegans* in the control and low Se treatments. However, the activity frequency of *C. elegans* in the high Se treatments (5 mg/kg and 10 mg/kg) decreased significantly, only 9.00 ± 0.71 and 9.22 ± 0.67 times per minute, which was evidently reduced by 23.6% and 21.73%. *Acrobeloides* sp. and *C. elegans* showed a similar change trend in activity frequency under different Se exposure concentrations. The activity frequency of *Acrobeloides* sp. on the first day in the control group was 8.11 ± 0.6 times per minute. A Se exposure concentration of 0.4 mg/kg had no significant effect on nematodes activity frequency. In contrast, activity frequency of *Acrobeloides* sp. increased strikingly to 9.33 ± 1.00, 12.00 ± 1.00, and 13.89 ± 1.05 times per minute when Se exposure concentration increased to 3 mg/kg, 5 mg/kg, and 10 mg/kg, respectively. On the seventh day, the activity frequency of *Acrobeloides* sp. in the low Se treatments (0.4 mg/kg and 3 mg/kg) did not change significantly, while in the high Se treatments, the activity frequency was only 7.56 ± 0.88 and 7.56 ± 1.33 times per minute, which was 14.96% lower than that in the control group.

**Figure 4:**
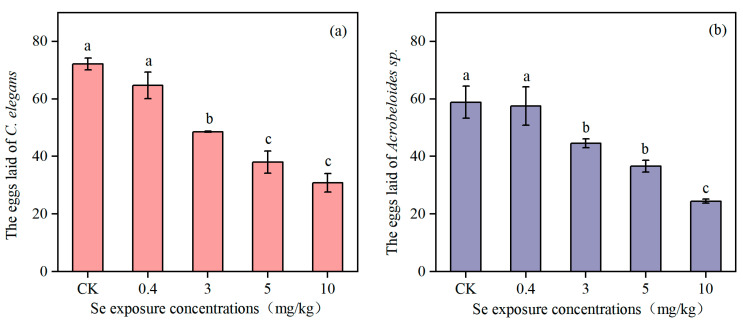
The eggs laid of *C. elegans* (**a**) and *Acrobeloides* sp. (**b**) under Se exposure (*n* = 3). Lowercase letters (a, b, c) differed to indicate significant differences between groups (*p* < 0.05).

Se exposure had a significant effect on the number of eggs laid of both *C. elegans* and *Acrobeloides* sp. As can be seen from Figure 4, the number of eggs laid in *C. elegans* was greater than that in *Acrobeloides* sp. The number of eggs laid by *C. elegans* in the control group was 72.17 ± 2.04, while the number of eggs laid by *Acrobeloides* sp. was only 58.84 ± 5.62. There was no significant effect of 0.4 mg/kg Se exposure on the number of eggs laid by both species of nematodes. When the Se content reached 3 mg/kg, 5 mg/kg, and 10 mg/kg, the number of eggs laid by both species of nematodes decreased significantly, being 48.54 ± 0.2, 38.02 ± 3.90, and 30.84 ± 3.24, respectively, which reduced by 32.73%, 47.31%, and 57.26%. The number of eggs laid of *Acrobeloides* sp. was 44.58 ± 1.53, 36.6 ± 2.06, and 24.43 ± 0.70, respectively, which declined by 24.24%, 37.80%, and 58.48% compared with the control group.

### 3.3. Effects of Se on Antioxidant Indexes of Nematodes

**Figure 5:**
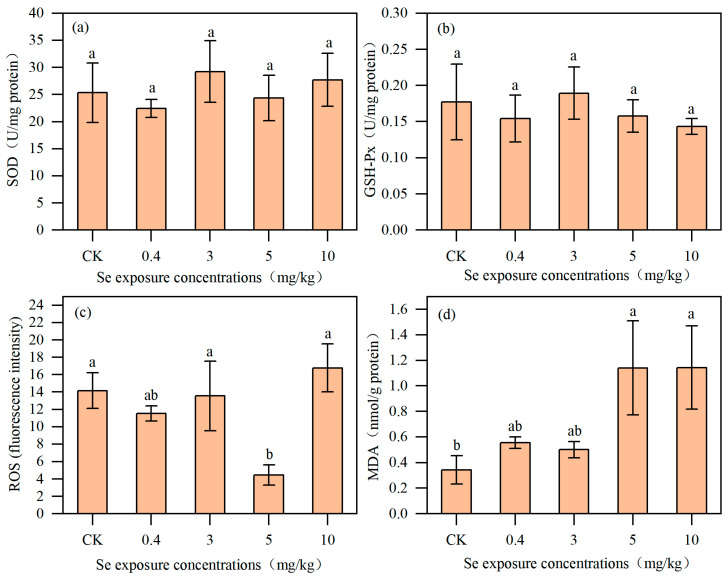
The SOD activity (**a**), GSH-Px activity (**b**), ROS level (**c**), and MDA contents (**d**) in *C. elegans* under Se exposure (*n* = 3). Lowercase letters (a, b) differed to indicate significant differences between groups (*p* < 0.05).

There was no significant difference in SOD activity in *C. elegans* between the control group and the Se exposure groups (Figure 5), but the highest value was reached (29.23 ± 5.69 U/mg protein) at 3 mg/kg Se treatment. Similarly, GSH-Px was not significantly affected by Se exposure contents. Compared with the control group, there was no significant change in ROS level in *C. elegans* at low Se concentrations (0.4 mg/kg and 3 mg/kg). However, ROS level was significantly lower at 5 mg/kg before returning to the control levels in 10 mg/kg treatment. The basal value of MDA content of *C. elegans* in the control group was 0.34 ± 0.11 nmol/mg protein. The MDA content in *C. elegans* remained unchanged at low Se concentrations (0.4 and 3 mg/kg) but increased significantly to 232.29% and 233.07% of the control level at high Se concentrations (5 and 10 mg/kg), respectively.

**Figure 6:**
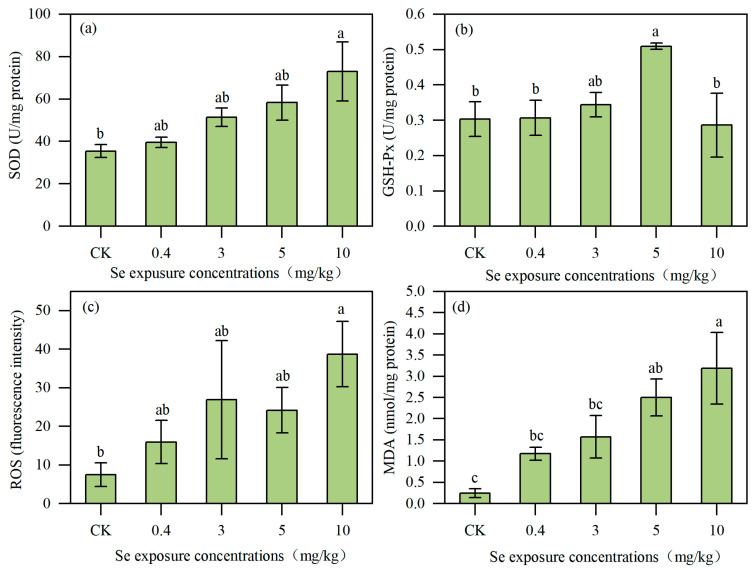
The SOD activity (**a**), GSH-Px activity (**b**), ROS level (**c**), and MDA contents (**d**) in *Acrobeloides* sp. under Se exposure (*n* = 3). Lowercase letters (a, b, c) differed to indicate significant differences between groups (*p* < 0.05).

In *Acrobeloides* sp. (Figure 6), the SOD activity was the lowest in the control group (35.45 ± 1.01 U/mg protein), and it increased under 0.4, 3, and 5 mg/kg treatments but not significantly. When it comes to the 10 mg/kg treatment, the activity of SOD increased significantly to 72.98 ± 13.99 U/mg protein, which was 105.87% higher than that of the control level. The activity of GSH-Px in the control group was 0.30 ± 0.05 U/mg protein, and there was no significant effect on low Se exposure concentrations (0.4 mg/kg and 3 mg/kg). The activity of GSH-Px increased to the peak value at 5 mg/kg and then fell back to the control levels at 10 mg/kg. The change in ROS was similar to that of SOD, which increased with the growth of Se content, and increased by 417.09% at 10 mg/kg treatment compared with the control group. The MDA content was 0.25 ± 0.10 nmol/mg protein in the control group, and it increased steadily with the increase in Se concentrations, reaching the highest value of 3.19 ± 0.84 nmol/mg protein in the 10 mg/kg treatment, which was 11.76 times higher than that of the control group.

## 4. Discussion

### 4.1. Se Application Threshold Based on Toxicological Response of Nematodes

Locomotion and reproductive capacity are the most important parameters for evaluating the ecotoxicological effects of chemicals on nematodes. The results showed that Se exposure had a content-dependent effect on the locomotion and reproductive ability of both *C. elegans* and *Acrobeloides* sp. Low Se concentrations had no significant effect, whereas high Se concentrations inhibited both activity frequency and reproduction. Similarly to the results of this study, the studies of Rohn et al. (2018) and Morgan et al. (2010) also noted that Se exposure reduced the locomotion and reproductive ability of nematodes [13,28]. Given its unique functions, Se can adversely affect reproduction, which potentially leads to a reduction in animal population [29,30]. In their investigation of Se toxicity throughout the life cycle of *C. elegans*, Li et al. (2014) reported that exposure to Se(IV)-contaminated environments posed significant reproductive risks to the nematode [31]. However, Li et al. (2011) reported a different finding, suggesting that the number of eggs laid by *C. elegans* first increased and then decreased as the Se exposure contents rose [14]. This variance may be the differences in the Se concentration gradient across studies. When the gradient of Se concentrations is too large, the promotive effect of Se on the locomotion and reproductive ability of nematodes can be overlooked, thereby resulting in only the inhibitory effect being observed. In this study, it can be seen that on the first day exposure of Se, the activity frequency of the nematodes increased with the increase in Se exposure concentration (Figure 2). This may be because motor neurons account for about one-third of all neurons in *C. elegans*, with 116 neurons forming neuromuscular connections with head or body muscles. The locomotion behavior of *C. elegans* is often used as an indicator of the basic nervous system function [32]. During short-term exposure to high concentrations of Se, the activity frequency of nematodes increases, whereas it decreases under longer-term exposure. This hormetic response indicates that Se initially acts as a neuro-excitatory agent, first stimulating the motor nervous system before subsequently exerting a toxic inhibitory effect. Laser ablation for killing specific neurons in *C. elegans* can be used to explore how Se affects the motor nervous system to inhibit or stimulate locomotion [33]. The number of eggs laid decreased significantly when *C. elegans* was exposed to 3 mg/kg or higher Se concentrations, and the activity frequency on the seventh day reduced significantly when exposed to 5 mg/kg or higher Se concentrations. There was no beneficial effect of Se observed, indicating that the benefits of Se may occur in the range of 0.4–3 mg/kg. *Acrobeloides* sp. and *C. elegans* had the same response to Se. Excessive Se can accumulate in the soil and harm the nearby ecosystem due to its limited uptake by crops.

Song et al. (2022) proposed a soil Se threshold for the effective concentration of soil nematodes and confirmed the negative effects of excessive Se on soil nematodes [34]. Their study provides insights for balancing Se biofortification in rice with the protection of the soil nematodes community. In this study, 3 mg/kg was initially obtained as the critical threshold for Se toxicity to nematodes by controlling the culture conditions. However, the farmland environment is extremely complex, and there are significant differences in the background concentration of Se in the actual soil. The Se concentration in soils worldwide is highly variable, typically ranging from 0.01 to 2 mg/kg, with a large proportion of soils being Se-deficient. This widespread deficiency is a key reason for the application of selenium fertilizers in agriculture. In the process of Se biofortification, it is necessary to determine the background concentration of Se in soil and the amount of Se application should be continuously adjusted to avoid toxic effects on soil organisms from the excessive application of Se fertilizer, and to provide a reliable basis for its safe application in the field.

### 4.2. Acrobeloides *sp.* Is a Suitable Bioindicator to Se

Currently, *C. elegans* is the most widely used soil nematode for ecotoxicological testing [35]. There are few studies investigating the uptake and accumulation of Se in soil nematodes, and Rohn et al. (2018) reported that under acute selenite exposure, the total Se content in *C. elegans* increased with the growth of Se exposure concentrations [13]. Similarly, the total Se content in nematodes in this study also increased with the increase in Se exposure concentration, and the Se accumulation ability of *Acrobeloides* sp. was stronger. Compared with *C. elegans*, *Acrobeloides* sp. has a smaller body size, lower activity, and a longer generation time, which may be the key factors contributing to its stronger Se accumulation [36]. SeMet and SeCys_2_ were the main Se species, indicating that both *C. elegans* and *Acrobeloides* sp. could convert inorganic Se in the environment into organic Se for storage. In addition, many studies have shown that there are some differences in metal bioaccumulation and sensitivity of different nematodes [37,38,39]. On the seventh day of Se exposure, the activity frequency of the nematodes decreased with increasing Se concentration. At a Se concentration of 5 mg/kg, the activity frequency of *C. elegans* and *Acrobeloides* sp. decreased by 23.58% and 15.00%, respectively. However, from Pearson correlation analysis (Table 2), the activity frequency and the eggs laid in *Acrobeloides* sp. were negatively correlated with the total Se content and most Se species (SeCys_2_, SeMet and Se^6+^). This correlation was stronger than those observed in *C. elegans*. The results may indicate that the internal response of *Acrobeloides* sp. to Se was stronger than that of *C. elegans*. In contrast to *C. elegans*, *Acrobeloides* sp., an indigenous nematode, is both abundant and widely distributed in farmland soils, making it a more suitable bioindicator for Se sensitivity in agricultural ecosystems. Soil nematodes have been widely recognized as effective bioindicators for assessing soil quality and environmental pollution, with their species-specific sensitivities compared under controlled laboratory conditions [38,40]. Given the distinct advantages of *Acrobeloides* sp., future studies should focus on the ecotoxicology and molecular mechanism of *Acrobeloides* sp. to better assess soil ecosystem health.

### 4.3. Inhibitory Effect of Se-Induced Oxidative Stress Mechanism on Nematodes

Pearson correlation revealed the correlation between the total Se content or different speciation and antioxidant indexes in *C. elegans* and *Acrobeloides* sp. (Table 2). In *C. elegans*, the total Se content, SeCys_2_ and Se^6+^ were positively correlated with the contents of MDA; the Pearson correlation coefficients were 0.61, 0.72 and 0.54, respectively (*p* < 0.05). In *Acrobeloides* sp., the total Se content, SeCys_2_, SeMet, and Se^6+^ were positively correlated not only with MDA content (*r* = 0.84, 0.67, 0.77, 0.76, *p* < 0.05), but also with SOD content (*r* = 0.91, 0.69, 0.86, 0.81, *p* < 0.05).

Oxidative stress is defined as an imbalance between oxidants and antioxidants, resulting in the interruption of redox signaling and control and molecular damage [41]. In *C. elegans*, Se exposure had no significance on SOD and GSH-Px. ROS decreased only at 5 mg/kg of Se but increased at 10 mg/kg. Notably, the most significant effect was observed on MDA content, which increased significantly at Se concentrations ≥5.0 mg/kg compared to both the control and low Se exposure groups. As an indicator of lipid peroxidation, MDA content can reflect the degree of lipid peroxidation in a cell membrane and the intensity of stress response [42]. There was no change in the activity of antioxidant enzymes while the MDA content increased, indicating that high Se exposure aggravated oxidative stress of *C. elegans* and accelerated lipid peroxidation of cell membranes.

In *Acrobeloides* sp., with the increase in the Se exposure contents, the SOD activity, ROS, and MDA contents rose. The possible reason is that under low Se exposure, the ROS level in *Acrobeloides* sp. is low, and the correspondingly lower SOD activity helps maintain an appropriate ROS balance. Under high Se exposure, *Acrobeloides* sp. produces a large amount of ROS, resulting in cellular lipid peroxidation and increased MDA content. To counteract the high Se stress and restore redox balance, *Acrobeloides* sp. enhanced its SOD activity. Meanwhile, the proportion of Se^6+^ in *Acrobeloides* sp. decreased with the increase in Se concentration (Figure 1). It suggested that *Acrobeloides* sp. can reduce the toxic effect by converting more exogenous inorganic Se into organic Se despite high Se accumulation. With the increase in Se exposure concentrations and reproduction, the activity frequency of these two nematodes decreased along with the imbalance of the antioxidant system and the growth of oxidative stress response.

Morgan et al. (2010) and Boehler et al. (2014) found that Se-induced impairments in nematode growth and locomotion were accompanied by increased ROS levels and oxidoreductase expression [28,43]. Based on these findings, they proposed that Se may inhibit the growth, reproduction, and locomotion ability of *C. elegans* through oxidative stress mechanism. In this study, the SOD activity and MDA content of nematodes were positively correlated with the total Se and the content of each Se species (*p* < 0.05), which further proved their conclusion. The result suggested that the inhibitory effect of Se on nematodes was mediated by Se-induced oxidative stress mechanism, a process influenced by both the total Se content in nematodes and the specific Se speciation.

**Figure 7:**
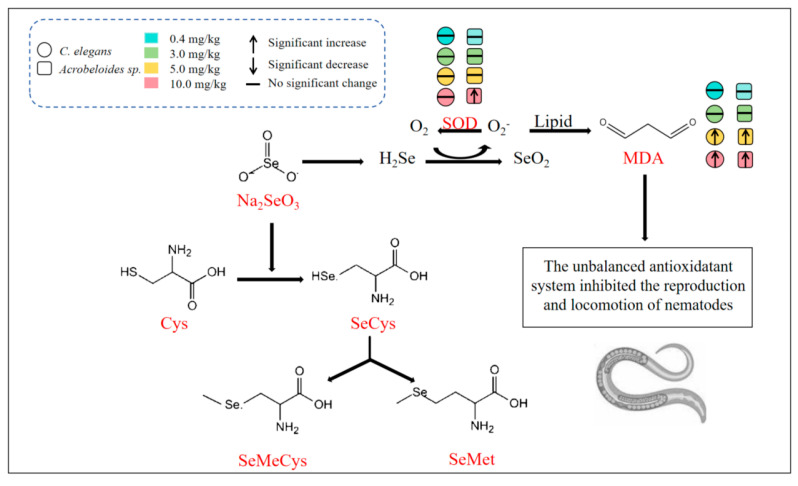
Schematic diagram of mechanism of Se’s toxic effects on nematode.

As shown in Figure 7, nematodes would be subjected to different effects when exposed to different concentrations of Na_2_SeO_3_. The intake of low Se concentrations (≤3 mg/kg) leads to the incorporation of Se into cysteine, forming SeCys biosynthesis [44]. Subsequently, the SeCys is metabolized to MeSeCys and SeMet. High concentrations of Na_2_SeO_3_ (≥5 mg/kg) are metabolized by glutathione (GSH) to hydrogen selenide (H_2_Se) via selenodiglutiathione and glutathionylselenol intermediates [45]. H_2_Se is then oxidized to Se dioxide (SeO_2_). Simultaneously, superoxide radicals (O_2_^−^), a type of ROS, are generated. The overproduction of ROS induces DNA and protein damage and lipid peroxidation, leading to increased MDA content [46]. This oxidative damage disrupts the antioxidant system and ultimately inhibits the nematode reproduction and locomotion.

## 5. Conclusions

The study analyzed the total Se content and Se speciation in nematodes after short-term exposure; investigated the effects of Se on the locomotion and reproduction capacity of *C. elegans* and *Acrobeloides* sp.; and verified the effects of Se on nematode via oxidative stress mechanism. The results showed that the total Se content in both nematodes increased with increasing Se exposure concentrations, and that *C. elegans* had a lower Se-accumulating ability than *Acrobeloides* sp. When the Se exposure concentrations ≥ 3 mg/kg, the reproduction of both *C. elegans* and *Acrobeloides* sp. was decreased significantly. When the Se exposure concentrations ≥ 5 mg/kg, the activity frequency of these two species nematodes also reduced. These results indicate that high Se exposure concentrations had inhibitory effects on the reproduction and locomotion capacity of both nematodes. There were differences in the distribution of Se species between the two nematodes, but SeMet and SeCys_2_ were the main Se species. Analysis of antioxidant indices showed that exposure to high Se concentrations (≥5 mg/kg) significantly increased the MDA content in *C. elegans*. Conversely, in *Acrobeloides sp*., significant increases were observed in SOD activity, ROS level, and MDA content. Pearson Correlation Analysis also confirmed that the total Se content and various Se species were positively correlated with the antioxidant enzyme activity and peroxide product content in nematodes, indicating that Se inhibits nematodes by inducing oxidative stress. This study confirms that the indigenous *Acrobeloides* sp. is an excellent organism for studying the ecotoxicological effects of Se. These results suggest that future studies should pay more attention to the response of indigenous organisms to trace elements. The results of this study provide important information for evaluating the soil ecotoxicity of Se. However, given the complexity of the soil environment, further long-term exposure experiments are necessary to assess the potential effects of Se on nematode community structure and soil ecosystem function.

## Figures and Tables

**Table 1:** Se contents in *C. elegans* and *Acrobeloides* sp. (*n* = 3).

Se Exposure Concentration (mg/kg)	Se Content in *C. elegans* (μg/mg Protein)	Se content in *Acrobeloides* sp. (μg/mg Protein)
Control (0)	0.06 ± 0.01 c	0.34 ± 0.15 c
0.4	0.78 ± 0.18 b	0.87 ± 0.14 c
3	4.78 ± 1.28 a	6.08 ± 2.13 bc
5	5.04 ± 0.85 a	9.77 ± 3.55 ab
10	5.70 ± 2.63 a	15.26 ± 2.91 a

Lowercase letter a, b, c differently indicates significant differences between groups (*p* < 0.05).

**Table 2:** Pearson correlations between different Se Species contents or total Se contents with antioxidant indexes in *C.elegans* and *Acrobeloides* sp.

		Activity Frequency on Seventh Day	Laid Eggs	SOD	GSH-Px	ROS	MDA
*C. elegans*	Total Se content	−0.80 *	−0.87 *	0.10	−0.11	−0.25	0.61 *
	SeCys_2_	−0.92 *	−0.86 *	0.04	−0.15	−0.42	0.72 *
	SeMet	0.13	−0.09	0.12	0.12	0.06	−0.22
	SeMeCys	0.15	−0.06	0.13	0.15	0.03	−0.26
	Se^6+^	−0.47	−0.58 *	0.07	−0.18	0.31	0.54 *
*Acrobeloides* sp.	Total Se content	−0.78 *	−0.90 *	0.91 *	0.23	0.59	0.84 *
	SeCys_2_	−0.63 *	−0.64 *	0.69 *	0.54	0.51	0.67 *
	SeMet	−0.72 *	−0.88 *	0.86 *	0.05	0.52	0.77 *
	SeMeCys	0.53 *	0.51	−0.35	−0.23	−0.19	−0.22
	Se^6+^	−0.68 *	−0.77 *	0.81 *	0.42	0.60	0.76 *

* indicates significant correlation (*p* < 0.05).

## Data Availability

Researchers wishing to access the data used in this study can make a request to the corresponding authors: songjp@ahstu.edu.cn and xbyin@ahstu.edu.cn.

## Supplementary Materials

The following supporting information can be downloaded at: https://www.mdpi.com/article/10.3390/toxics13100870/s1. Figure S1. Instrument calibration curves for SeCys_2_ (A), SeMeCys (B), Se^4+^ (C), SeMet (D), and Se^6+^ (E). Figure S2. Chromatogram of five Se species’ mixed standard samples.
