# Supplementary material for: Na2SeO3 Exposure Inhibits Locomotion and Reproduction via Oxidative Stress Mechanism in Bioindicators C. elegans and Acrobeloides sp
Source: Toxics. 2025 Oct 13;13(10):870. doi: 10.3390/toxics13100870 (PMC12567610; doi:10.3390/toxics13100870)
Supplement: Supplementary file 1 [file toxics-13-00870-s001.zip › toxics-3860946-supplementary.pdf]

Supplementary Materials

The five selenium species used in this experiment are as follows: $SeCys_2$ (>99%, purchased from Tokyo Chemical Industry), SeMet (>98%, purchased from AOROS Organics), SeMeCys (>98%, purchased from AOROS Organics), Se^{4+} and Se^{6+} (chromatographically pure, purchased from China National Pharmaceutical Group and Chemical Reagent Co., Ltd.). First, the standard samples of five selenium species were prepared at a concentration of 1000 mg/mL (expressed as selenium) and stored at -20°C. Before instrumental analysis, the stock solutions of the five Se species were diluted to 5 μg/L, 20 μg/L, 50 μg/L, 100 μg/L and 200 μg/L with 18.2 MΩ/cm ultrapure water. The instrument calibration curves for each selenium species were established, and once the correlation coefficient was close to 1, the analysis of test samples was determined. The resulting calibration curves of five Se species are shown in **Figure S1**.

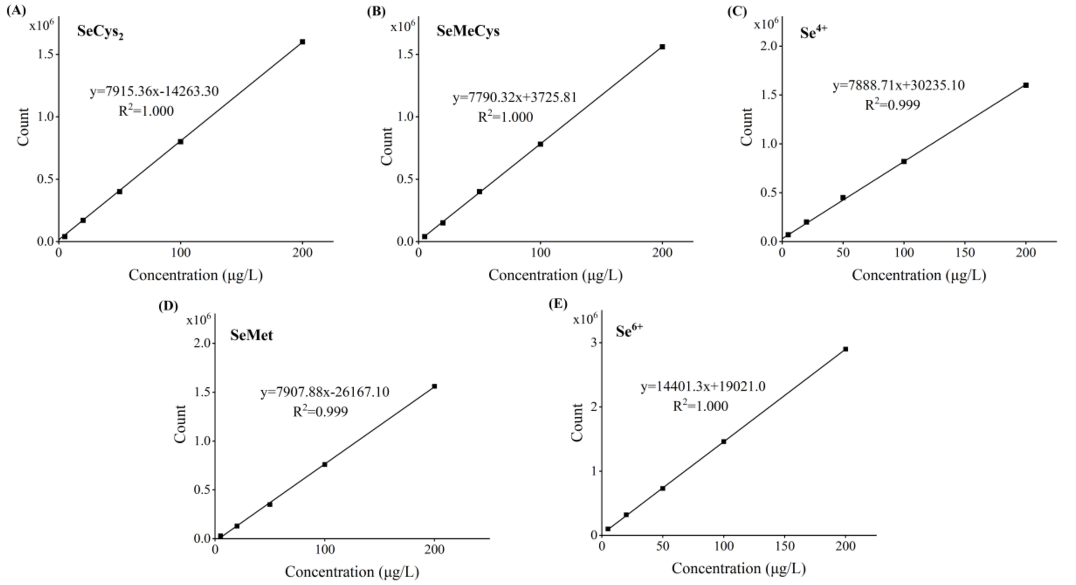

Figure S1. Instrument calibration curves for $SeCys_2$ (A), SeMeCys (B), Se^{4+} (C), SeMet (D), and Se^{6+} (E).

Using 1000 mg/mL (expressed as selenium) stock solution of five Se species standard samples, a mix of five selenium species standard solutions were prepared. The mixed standard solution was gradually diluted to a concentration of 100 μg/L. The mixed standard samples were analyzed using HPLC-ICP-MS to confirm the identity and separation of the peaks. The chromatogram of the five Se species' mixed standard

samples (100 μg/L, expressed as Se content) is shown in **Figure S2**.

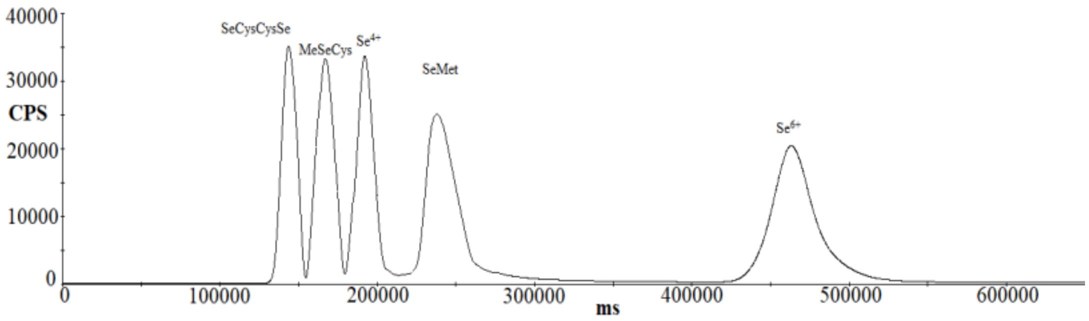

Figure S2. Chromatogram of five Se species' mixed standard samples.
